# Notes and News

**Published:** 1904-01-30

**Authors:** 


### Notes and News.

The late Mrs. Mary Elizabeth Turnbull has bequeathed £2,000 each to the Devonshire Hospital, Buxton, and the Children's Hospital, Paddington Green.

A petition is being signed by the registered medical practitioners of Great Britain to the central educational authorities of the kingdom urging that teaching in hygiene should be made compulsory in the public elementary and secondary schools.

The awards amounting to £200 from Mr. Hickman and the Royal Free Hospital for the discovery of Miss Hickman's body have been paid. Part of the sum has been divided between the boys concerned in finding the body, but the majority has been formed into a trust fund for them for their future benefit. Special contributions are being made to refund to the hospital the £100 it has paid.

Advantage might be taken of the occasion of the Medical Congress at Buenos Ayres in April for medical men interested in the study of tropical diseases, to take an interesting holiday. During the Congress important communications will be made on these subjects. Numerous other subjects will, of course, be under discussion, and a hygienic exhibition will be opened after the close of the meetings.

Free public lectures on tuberculosis and its remedies are given in Germany by the Committee for the erection of sanatoria for consumption. Our Society for the Prevention of Tuberculosis might follow suit with advantage. The early diagnosis of consumption is of so much importance in relation to its cure that if the lectures did no more than assist the public to detect the presence of the disease, they would be doing good work.

The late Mr. Edward Wilkinson has bequeathed £500 to the National Hospital for the Paralysed and Epileptic.

The Director of the Plague Laboratory of the Russian Imperial Institute of Experimental Medicine has died of the plague through experimenting with plague cultures. Anti-plague serum injections were made with no avail. Those in contact with him were at once subjected to the injection, and the laboratory has been isolated.

The small-pox epidemic at Glasgow shows no diminution. The experience of several members of the staff of the small-pox hospital serve to illustrate the necessity of securing complete vaccination before a risk of infection is taken. It is a popular idea that abortive vaccination is an indication that the vaccinated person has no tendency to small-pox. But this is certainly not the case.

The Church of England Temperance Society have opened a Home at Walpole, in Norfolk, for the after care of inebriates. The society intend to give the project one year's experiment. They have appealed for the small sum of £150 for this purpose, but have only received £97. The experiment is well worthy of encouragement, and the funds asked are so small they should certainly be forthcoming without delay.

Every sensible person must regret the introduction of measures having a pauperising tendency, but there are circumstances where the objection is outweighed by the good results likely to follow. Therefore we feel our sympathy goes with the authorities of the Manchester Voluntary Schools, who now provide the poorest of the children with dinner during the winter. Every precaution is taken to prevent abuse, and the benefit to the children many of whom have never had a regular meal in the middle of the day must be enormous. It would be useful were the weights of the children recorded, as there can be little doubt that a marked improvement in their condition would be shown.

A school for cripples, similar in aim to the one at Copenhagen lately described in The Hospital, has been started at Paulton's Square, Chelsea, under the direction of Sister Mary. The intention of the school is to bring up cripples to earn their own living at such trades as their limitations permit them to attempt. Boys will be taught tailoring and shoemaking, and arrangements have now been made to teach tapestry weaving to girls. Unfortunately, many of the children, whether their previous life has been passed at home or in hospital, are woefully illiterate at an age when their elementary education should be far advanced, nor have most of them ever been taught in any way to make use of their hands. This, added to the fact that a good many of the pupils are tuberculous, delicate creatures, unable to stand prolonged exertion, makes the task of teaching them very uphill work. But the results of the Danish school, which enjoys the patronage of the Royal Family of the country, including Queen Alexandra, shows how much can be accomplished with very unpromising material.

